# Unraveling Immune-Related lncRNAs in Breast Cancer Molecular Subtypes

**DOI:** 10.3389/fonc.2021.692170

**Published:** 2021-05-31

**Authors:** Carolina Mathias, João Carlos Degraf Muzzi, Bruna Borba Antunes, Daniela F. Gradia, Mauro A. A. Castro, Jaqueline Carvalho de Oliveira

**Affiliations:** ^1^ Department of Genetics, Federal University of Parana, Post-graduation Program in Genetics, Curitiba, Brazil; ^2^ Bioinformatics and Systems Biology Lab, Federal University of Parana (UFPR), Polytechnic Center, Curitiba, Brazil; ^3^ Immunochemistry Laboratory (LIMQ), Federal University of Parana, Post-graduation Program in Microbiology, Parasitology and Pathology, Curitiba, Brazil; ^4^ Instituto de Pesquisa Pelé Pequeno Príncipe, Oncology Division, Curitiba, Brazil

**Keywords:** immune response, MEG3, LINC01871, EBLN3P, LINC02613, XXYLT1-AS2

## Abstract

Breast cancer (BRCA) is the most leading cause of cancer worldwide. It is a heterogeneous disease with at least five molecular subtypes including luminal A, luminal B, basal-like, HER2-enriched, and normal-like. These five molecular subtypes are usually stratified according to their mRNA profile patterns; however, ncRNAs are increasingly being used for this purpose. Among the ncRNAs class, the long non-coding RNAs (lncRNAs) are molecules with more than 200 nucleotides with versatile regulatory roles; and high tissue-specific expression profiles. The heterogeneity of BRCA can also be reflected regarding tumor microenvironment immune cells composition, which can directly impact a patient’s prognosis and therapy response. Using BRCA immunogenomics data from a previous study, we propose here a bioinformatics approach to include lncRNAs complexity in BRCA molecular and immune subtype. RNA-seq data from The Cancer Genome Atlas (TCGA) BRCA cohort was analyzed, and signal-to-noise ratio metrics were applied to create these subtype-specific signatures. Five immune-related signatures were generated with approximately ten specific lncRNAs, which were then functionally analyzed using GSEA enrichment and survival analysis. We highlighted here some lncRNAs in each subtype. LINC01871 is related to immune response activation and favorable overall survival in basal-like samples; EBLN3P is related to immune response suppression and progression in luminal B, MEG3, XXYLT1-AS2, and LINC02613 were related with immune response activation in luminal A, HER2-enriched and normal-like subtypes, respectively. In this way, we emphasize the need to know better the role of lncRNAs as regulators of immune response to provide new perspectives regarding diagnosis, prognosis and therapeutical targets in BRCA molecular subtypes.

## Introduction

Breast cancer (BRCA) is a molecular and histological heterogeneous disease with at least five intrinsic molecular subtypes (1, 2). Based on gene expression, BRCA can be mainly classified into luminal A (LumA), luminal B (LumB), HER2-enriched (Her2), basal-like (Basal), and normal-like (Normal) (3, 4). These subtypes have a distinct prognosis and also differ according to therapeutic response. LumA and LumB tumors respond well to hormonal interventions, while HER2+ tumors respond effectively when anti-HER2 therapy is used (5). Basal tumors are very aggressive and associated with the shortest survival times, with no current molecular-based targeted therapies available (6).

Immunotherapy brought a new line of action in cancer care; however its response varies across cancer types and patients. The immune system response in the tumor microenvironment may help to guide immunotherapy drug discovery and clinical decisions (7). In general, tumors more responsive to immune checkpoint inhibitors are related to high levels of leukocyte fraction in the tumor microenvironment (8). Besides gene expression differences in BRCA molecular subtypes, they differ significantly concerning the composition of cells that form the tumor microenvironment, especially the immune system’s cells. A substantial proportion of natural killer cells and neutrophils have been found in luminal tumors. In contrast, in these tumors, cytotoxic T cells (T CD8+) and naïve and memory T cells are found less frequently. In BRCA Basal tumors, T regs, associated macrophages 2, and activated mast cells form a significant portion of the immune infiltrate cells. The immune infiltrate composition is not widely described in the Her2 subtype. It is known that it is mainly formed by dendritic cells, mast cells, γδ T lymphocytes, T regs and neutrophils (9).

A landscape of tumors’ immune microenvironment was characterized from immunogenomics data by Thorsson and colleagues (8). In this study, using an integrated analysis, they could classify solid tumors (from The Cancer Genome Atlas) into six major immune subtypes, which they called C1-C6. These subtypes have distinct immune signature sets, which could also be related to prognosis. C1 (wound healing) exhibited elevated expression of angiogenic genes, a high proliferation rate, and a Th2 cell bias to the adaptive immune infiltrate. C2 (IFN-γ dominant) had a strong T CD8+ signal, the greatest TCR diversity, and a high proliferation rate. C3 (inflammatory) was the subtype that presented high Th17 and Th1 genes and low to moderate tumor cell proliferation. C4 (lymphocyte depleted) displayed a more prominent macrophage signature with Th1 suppressed and high M2 response. C5 (immunologically quiet) was enriched by brain tumors and exhibited the lowest lymphocyte and most increased macrophage responses. Finally, C6 (TGF-β dominant) displayed the highest TGF-β signature and a high lymphocytic infiltrate with an even distribution of type I and type II T cells (8).

According to this approach, BRCA could be classified into five subtypes (C1, C2, C3, C4 and C6), being C2 (n=345) the most representative subtype, followed by C1 (n=320). Immune subtypes were also described according to BRCA molecular subtypes, and as expected, the subtypes varied significantly according to these immune groups. For example, LumA was more representative of the C1 subtype, while Basal samples of C2 (8).

Gene expression sets based on mRNAs were used for the classification and determination of molecular and immune subtypes. However, it is already known that the most abundant part of the human genome is not translated into proteins. These transcripts are organized in a class called “non-coding RNAs.” Non-coding RNAs can be classified into two major categories based on their size: small non-coding RNAs (<200 nucleotides) and long non-coding RNAs (>200 nucleotides) (10). LncRNAs are usually transcribed by RNA polymerase II, polyadenylated, and capped. They exhibit high tissue specificity and great regulatory versatility, acting at different gene expression regulation levels (11, 12).

Due to its high tissue specificity, lncRNAs can be evaluated as potential disease biomarkers, including BRCA (13–15). Based on this, we looked for molecular subtype specific lncRNAs signatures that could help differentiate the immune profiles described in Thorsson (8). These lncRNAs were also analyzed if impact the patient’s overall survival and progression free interval and were also investigated in differential expression and enrichment analysis to explore other possible biological roles of these lncRNAs in BRCA molecular and immune subtypes.

## Material and Methods

### Breast Cancer Immune Data

BRCA molecular and immune subtypes, leukocyte fraction, and survival information were downloaded from Thorsson et al. (8) **Supplementary Material**. According to samples’ barcode expression and immune type, data were integrated to perform the analysis. In **Supplementary Table 1**, we organized data according to breast cancer molecular and immune subtypes.

### Breast Cancer RNA-Seq Data

Log2 normalized FPKM RNA-Seq data from The Cancer Genome Atlas (TCGA) breast cancer cohort was downloaded from XenaBrowser (https://xenabrowser.net/datapages/), and primary tumor samples were selected and merged with Thorsson et al. (8) master table using patients’ barcode. The lncRNAs and protein-coding gene expression profiles were filtered from the RNA-Seq data using the R package biomaRt v 2.46.3 (16). For lncRNAs, when available, HGNC Symbol was used; otherwise, Ensembl gene name was used. All Ensembl and HGNC Symbols from lncRNAs used in this study are available in **Supplementary Table 2**. The non-tumoral samples were selected based on TCGA barcodes ending with 11A or 11B. The molecular BRCA subtypes were defined as described in Thorsson et al. (8) **Supplementary Material**, based on PAM50.

The expression profiles of immunomodulatory genes listed (8) (https://www.cell.com/cms/10.1016/j.immuni.2018.03.023/attachment/8d3ffc74-4db4-4531-a4ad-389dfc8bb7ec/mmc7.xlsx) previously were obtained from the gene expression matrix. Of the 75 immune modulators, only one (C10orf54) was not found in the expression matrix. For heatmap construction using *ComplexHeatmap* package (17), samples were displayed in columns and genes in the rows. Column-wise z-score was calculated for gene expression values, and maximum and minimum values were limited to +2 and −2 standard deviations, respectively. Samples were clustered within each BRCA subtype.

The BRCA lncRNAs expressions were further filtered above the first quartile for lncRNA expression sum, which means lncRNAs with expression sum above 8.04 log2 FPKM in the whole BRCA cohort (1,060 samples). Signal-to-noise ratio (SNR) was calculated for each molecular subgroup individually as follow:

$$SNR=\frac{\mu 1-\mu 2}{\sigma 1+\sigma 2}$$

being μ_1_ the mean of lncRNA expression in the group analyzed and μ_2_ the mean of lncRNA expression in the patients out of the group analyzed. σ refers to the standard deviation from the respective groups mentioned. We selected the lncRNAs above the SNR 0.95 quantile for each BRCA molecular subtype, that means the lncRNAs with higher expression in the subtype analyzed compared with the rest of the cohort. Venn diagram was constructed using *InteractiVenn* (http://www.interactivenn.net/) demonstrating the intersection of lncRNA between groups. Then, we calculated the SNR within each molecular subgroup based on the immune subtypes described previously (8). We considered for the analysis the immune groups with more than five patients in each molecular subtype. In this way, Basal and Her2 samples were divided into C1 and C2 subtypes; LumA and Normal into C1, C2, C3, C4, and C6 subtypes and LumB into C1, C2, C3, and C4 subtypes. After absolute SNR sum calculation, we selected lncRNAs considered in 0.98 quantile, which means the lncRNAs with the most significant variation within the immune subtypes for each molecular subgroup. For histogram construction, the absolute SNR sum was scaled using z-score. The 53 lncRNAs selected from this analysis are presented in **Supplementary Table 3**.

### Leucocyte Fraction Correlation

The lncRNAs expression was used to calculate Spearman correlation with the leucocyte fraction observed in each BRCA subtype, and the p-value was calculated with AS 89 algorithm (18) using cor. test function from stats R package v.4.0.4 (19) and adjusted by False Discovery Rate (FDR) method.

### Survival Analysis

Survival analysis was firstly performed using the *coxph* function available in the survival R package v3.2-10 (20) based on lncRNA expression for both Overall Survival (OS) and Progression-Free Interval (PFI) for each lncRNA individually in its respective BRCA molecular subgroup. The univariate Cox p-value for each lncRNA was calculated and adjusted by the FDR method; values below 10% FDR level were considered significant.

One lncRNA was selected on each BRCA subtype based on Cox results or Leukocyte Fraction correlation for further analysis. Kaplan-Meier was calculated, and patients were divided by the median lncRNA expression value in High Expression and Low Expression; p-value was calculated by log-rank test.

### Enrichment Analysis

The protein-coding genes expression profile was filtered for each molecular subgroup by patients’ barcode, and genes with zero sum expression were removed. Patients were divided by the median lncRNA expression value, and High versus Low Expression groups were used for SNR calculus. Genes were ordered by SNR value, and gene set enrichment analysis (GSEA) was inferred using fgsea R package v1.16.0 (21) with MSigDb v7.2 Hallmarks gene sets (22, 23) for 10,000 permutations.

## Results

### Immune Modulator Genes Expression Demonstrates Distinct Patterns Within BRCA Molecular Subtypes

Based on the gene expression profile related to immune response, Throsson et al. (8) analyzed over 10,000 TCGA samples, from which 1,087 were from BRCA samples and clustered them in six immune subtypes. We merged the barcodes with the gene expression matrix downloaded from XenaBrowser, remaining 1,060 BRCA primary tumor samples. The molecular classification as presented in Thorsson et al. (8) master table as TCGA Subtype was used, dividing the samples into five groups, LumA (n=499), LumB (n = 184), Basal (n=169), Normal (n=136) and Her2 (n=72). In **Supplementary Table 1**, we represented the number of samples according to each molecular subgroup’s immune subtype.

**Supplementary Figure 1** shows a distinct pattern of immune modulators gene expression in all molecular subtypes. In general, most genes seem to be upregulated in the same samples (columns of the heatmap) independent of its classification as an inhibitor or stimulator of the immune system. Overall, the immune activation seen in the gene expression follows the rising in Leukocyte Fraction and tends to group the C2 immune subtype. Simultaneously, the inverse is observed for a low expression pattern associated with low Leukocyte Fraction and C1 immune subtype. Basal and Her2 demonstrate a more apparent separation of C1 and C2. These two immune subtypes are the major representatives in these molecular groups; for instance, C1 and C2 represent 95.9% and 93.1% of all Basal and Her2 samples, respectively (**Supplementary Table 1**).

### SNR Highlights Specific lncRNAs for Each BRCA Molecular Subgroups Related to Immune Subtypes Distinction

**Figure 1:**
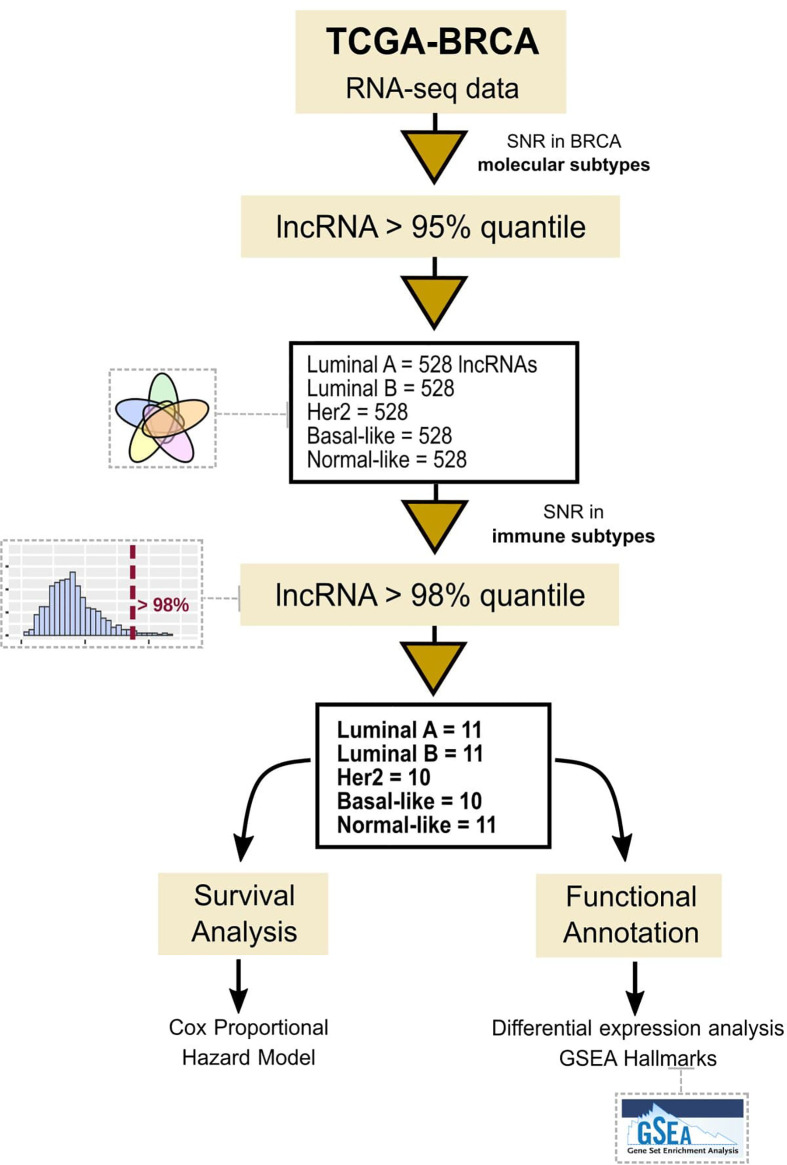
Workflow representation of the used methodology to study immune-related lncRNAs.

**Figure 2:**
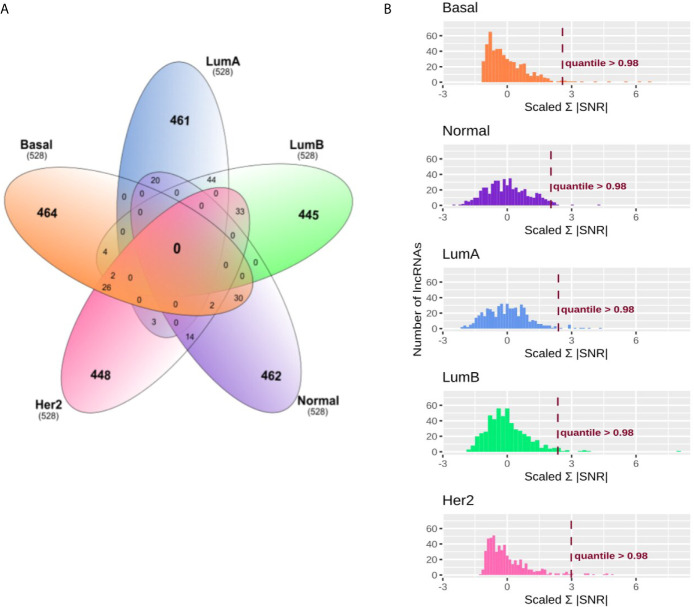
Immune related lncRNAs filter and selection in breast cancer (BRCA) molecular subtypes. **(A)** Venn diagram representing specific and shared immune related lncRNAs in breast cancer molecular subtypes. After filtering for 0.95 quantile in signal to noise ratio (SNR) for the BRCA molecular subtypes, 528 lncRNAs were selected for the next analysis. **(B)** Absolute SNR sum filter selection. SNR was calculated within each BRCA molecular subtype for the immune subtypes. Only groups with more than five patients were used. The histograms represent the amount of lncRNAs in each range of absolute SNR sum for the BRCA molecular subtypes. X-axis was scaled for z-score to allow comparison. The lncRNAs were filtered according to the 0.98 quantile as represented as the dashed line on the histograms.

The first step of our strategy is to search for lncRNAs that could be associated with immune subtypes in BRCA patients. For this, lncRNAs with greater distinct expression patterns among the five molecular subgroups were selected (**Figure 1**). After selecting the 0.95 quantile in SNR values and including the only 5% more differentially expressed in each subtype, we obtained 528 lncRNAs for each BRCA molecular subtype. **Figure 2A** shows how these lncRNAs were intersected between the five subgroups. None lncRNA was shared between all subgroups, and most of them (over 80%) were specific for each molecular subtype. The most significant intersections were seen between LumA and LumB (44 lncRNAs), LumB and Her2 (33 lncRNAs), Basal and Normal (30 lncRNAs) and Basal and Her2 (26 lncRNAs), which represents less than 10% of the 528 lncRNAs defined for each subgroup. Even when selecting the 0.90 quantile in SNR values, none lncRNA was shared between all molecular subtypes (**Supplementary Figure 2**) which shows that the SNR was able to distinguish specific lncRNAs for each molecular subtype.

Secondly, we calculated the absolute SNR sum for the immune subtypes for these 528 lncRNAs selected for each BRCA molecular subgroup. **Figure 2B** shows the distribution of absolute SNR sum for the lncRNAs. Z-score was calculated to allow comparison between groups. The five groups demonstrated different distributions, being Basal and Her2, characterized for most lncRNAs with slight variation between the immune subtypes, while LumA and Normal presented higher variation. After selecting the 0.98 quantile (**Figure 2B** and **Supplementary Table 3**), 11 lncRNAs remained, of which only one was shared between Her2 and Basal, the lncRNA KLHDC7B-DT (ENSG00000272666). This lncRNA was removed for further analysis as we looked for a specific lncRNA signature related to each BRCA molecular subgroup. Eleven specific lncRNAs were selected for LumA, LumB and Normal and ten for Her2 and Basal. All results for the first and the second SNR calculation as well as the quantile for each lncRNA is presented in **Supplementary Table 3**.

### Specific lncRNAs in BRCA Molecular Subgroups Are Associated With Immune Subtypes Differentiation

**Figure 3:**
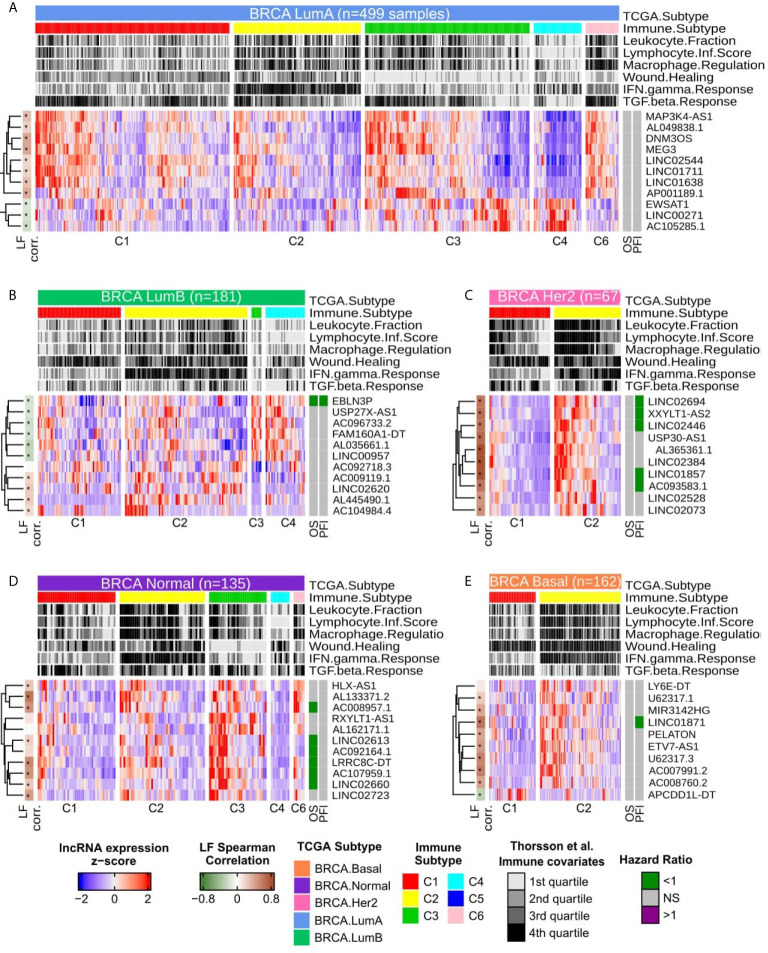
Heatmap with column-wise z-scores for immune related lncRNAs for each BRCA molecular subtype. For color gradient, maximum and minimum z-scores were set to +2 and −2 respectively. Each column represents a sample and were semi-supervised clustered within the immune subtypes. Immune subtypes groups with less than 5 patients are not represented as they were not used in SNR calculation. The top annotations present the molecular subtype with the number of samples, the immune subtype and the immune covariates as described by Thorsson et al. (8). The immune covariates are presented as quartiles in all TCGA-BRCA primary tumor samples. The left annotation shows the Spearman’s correlation for each lncRNAs with Leukocyte Fraction, being dark green for negative and brown for positive ρ values, asterisks represent Spearman’s correlation adjusted p-values values below 0.05. The right annotation represents the Hazard Ratio (HR) inferred by Cox Univariate analysis for Overall Survival (OS) and Progression Free Interval (PFI). Cox results with adjusted p-values greater than 0.1 are considered non significant and are colored in grey, otherwise, HR values above 1 are colored purple while HR values below 1 are colored in green. **(A)** LumA samples. **(B)** LumB samples. **(C)** HER2-enriched samples. **(D)** Normal-Like samples. **(E)** Basal-Like samples.

The remaining specific lncRNAs related to immune subtypes are presented in **Figure 3**; only immune subtypes with more than five patients are shown. These were the groups used for SNR analysis. For LumA, the lncRNAs clearly distinguished the C4 and C6 subtypes while demonstrating a mixed pattern in C1, C2 and C3. Nevertheless, EWSAT1, LINC00271 and AC105285.1 show higher activation in C3 than in C2, for example. No significant correlation with OS or PFI was observed for lncRNAs expression in Cox univariate analysis in LumA patients (**Figure 3A** and **Supplementary Table 4**).

For LumB (**Figure 3B**), a clear distinction can be seen between C3 and C4 in the gene expression pattern, while LINC02620 and mainly AL445490.1 showed a higher expression pattern in C3. Only EBLN3P correlated with a good prognosis in both OS and PFI for Cox univariate analysis (**Figure 3B** and **Supplementary Table 4**). In the Her2 samples (**Figure 3C**), all ten specific lncRNAs selected demonstrated a higher expression pattern in C2 and a strong positive correlation with Leukocyte Fraction. From the Cox univariate analysis, after p-values adjustment, half of the ten lncRNAs were associated with good prognosis in PFI, but none related to OS (**Figure 3C** and **Supplementary Table 4**).

In Normal (**Figure 3D**), all 11 lncRNAs were suppressed in the C4 subtype and, in general, showed a higher activation pattern in C3 and less evidently in C2. Despite C6 being represented by only six patients, HLX-AS1 and AL133371.2 appeared highly expressed in the C6 group. In Cox analysis, after p-adjustment, six from the 11 lncRNAs had a hazard ratio (HR) < 1 for OS, although none presented significant values for PFI. Finally, in the Basal group (**Figure 3E**), nine lncRNAs presented a higher expression in C2 and a lower expression in C1, while APCDD1L-DT presented an inverse pattern. Only LINC01871 showed a significant correlation with PFI, but not for OS (**Figure 3E** and **Supplementary Table 4**).

### lncRNAs Functional Annotations and Survival Analysis

We used MSigDb Hallmarks gene sets for GSEA analysis to infer possible biological roles associated with the specific lncRNAs expression (**Figure 3**); the results for all 53 lncRNAs are presented in **Supplementary Tables 5–9** and **Supplementary Figure 3**. We selected one lncRNA from each BRCA molecular subgroup to focus. For LumB, Her2, Basal and Normal, we chose the smallest p-value in Cox univariate analysis. As LumA did not present any significant p-values in Cox univariate analysis, we selected MEG3 as it showed a higher correlation with Leukocyte Fraction (Spearman’s ρ=0.4, p-adjusted < 2.2 × 10^−16^).

#### BRCA Basal-Specific lncRNAs Are Associated With Interferon Gamma Response and Allograft Rejection Gene Sets. LINC01871 Is More Expressed in Basal and Relates to Better OS and PFI

The GSEA analysis for the selected lncRNAs from BRCA Basal group revealed immune related gene sets, like Interferon Gamma Response, Allograft Rejection and Interferon Alpha Response, enriched in all ten lncRNAs, being negatively associated only with APCDD1L-DT (**Supplementary Table 5** and **Supplementary Figure 3A**), which was also the only one from the ten lncRNAs negatively related to Leukocyte Fraction (**Supplementary Table 4**).

**Figure 4:**
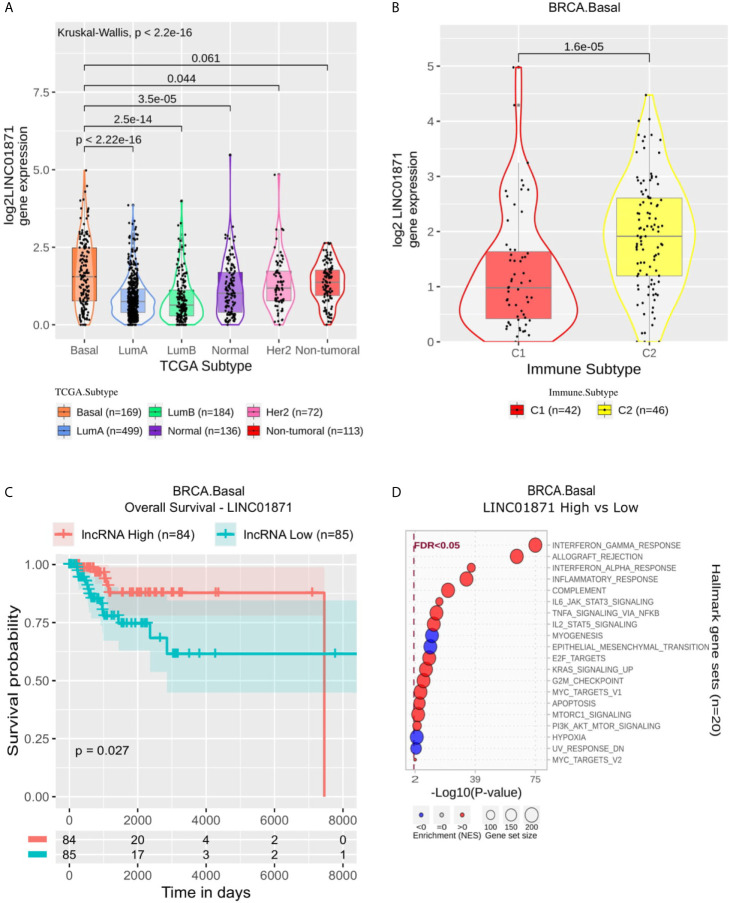
LINC01871 panel representation in basal-like subtype. **(A)** Box-plots representing LINC01871 expression among all breast cancer molecular subtypes. The Kruskal-Wallis test (p-value represented in the panel left top position) was used to differentiate expression between groups, followed by the Wilcoxon test (p-values represented emphasizing comparisons between subtypes). **(B)** Box-plot representing LINC01871 in C1 and C2 immune subtypes in basal-like patients. **(C)** LINC01871 overall survival curves. To define the two groups, LINC01871’s median expression classified the patients into “high” and “low” groups. Logrank p-value represented as 0.027. **(D)** Enrichment analysis using Hallmarks gene sets. Red circles refers to activation while blue circles to inactivation. In the X axis, a p-value scale is represented. The circle size varies according to the number of genes in the identified gene set.

Focusing on LINC01871, this lncRNA presented a higher expression in Basal than all other BRCA molecular subtypes (**Figure 4A**) and was mainly associated with immune activation, for instance, enriched in Interferon Gamma Response, Allograft Rejection, Interferon Alpha Response and Inflammatory Response gene sets (**Figure 4D**). Within Basal samples, it appears significantly suppressed in C1 (**Figure 4B**), while in Kaplan-Meier analysis, it was associated with better OS and PFI (**Figure 4C** and **Supplementary Figure 4A**).

#### BRCA Normal Specific lncRNAs Are Associated With Suppression of Proliferation Gene Sets. LINC02613 Is Suppressed in C4 and Relates to OS

**Figure 5:**
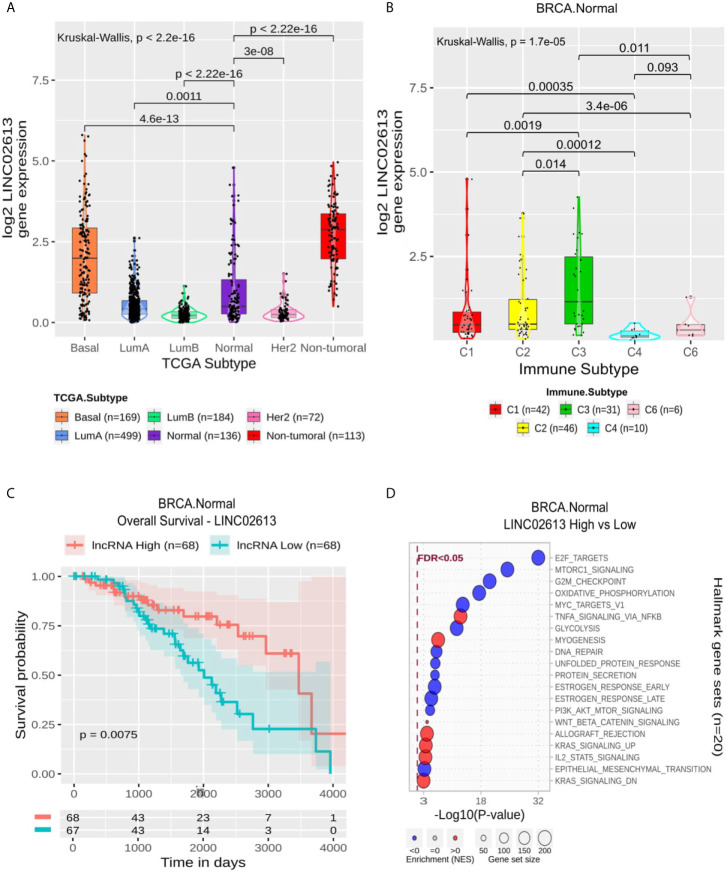
LINC02613 panel representation in normal-like subtype. **(A)** Box-plots representing LINC02613 expression among all breast cancer molecular subtypes. The Kruskal-Wallis test (p-value represented in the panel left top position) was used to differentiate expression between groups, followed by the Wilcoxon test (p-values represented emphasizing comparisons between subtypes). **(B)** Box-plot representing LINC02613 in C1,C2, C3, C4 and C6 immune subtypes in normal-like patients. **(C)** LINC02613 overall survival curves. To define the two groups, LINC02613’s median expression classified the patients into “high” and “low” groups. Logrank p-value represented as 0.0075. **(D)** Enrichment analysis using Hallmarks gene sets. Red circles refers to activation while blue circles to inactivation. In the X axis, a p-value scale is represented. The circle size varies according to the number genes in of the identified gene set.

The 11 BRCA Normal specific lncRNAs were related to the suppression of gene sets like G2M checkpoint, E2F and MYC targets associated with proliferation (**Supplementary Table 6** and **Supplementary Figure 3B**). Mainly AL133371.2, AC008957.1, LRRC8C−DT, AC107959.1, LINC02660 and LINC02723 presented immune-related gene sets positively enriched (Allograft Rejection, Coagulation, Complement, IL6 JAK STAT3 Signaling, Inflammatory, Interferon Alpha and Interferon Gamma Response). LINC02613 follows this enrichment pattern (**Figure 5D**). It is worth noting that Estrogen Response Early and Late gene sets appear suppressed only for this lncRNA and AC092164.1. Although its expression significantly differs from BRCA Normal to all other molecular subtypes (**Figure 5A**), it is more expressed in non-tumoral samples followed by BRCA Basal and Normal. At the same time, it seems suppressed in LumA, LumB and Her2. Within Normal samples, it is more expressed in C3 and significantly suppressed in C4 (**Figure 5B**) and correlates with a better OS (**Figure 5C**) but not in PFI (**Supplementary Figure 4B**).

#### LumA-Specific lncRNAs Relate to Epithelial-Mesenchymal Transition, Immune and Proliferation Gene Sets. MEG3 Is Suppressed in BRCA Compared With Non-Tumoral Samples

For LumA (**Supplementary Table 7** and **Supplementary Figure 3C**), the Epithelial-Mesenchymal Transition (EMT) module was enriched in all 11 lncRNAs, being EWSAT1, LINC00271, and AC105285.1 associated negatively with this module and the other eight lncRNAs positively. In general, this pattern was followed for the immune-related gene sets and the correlation with Leukocyte Fraction (**Figure 3** and **Supplementary Table 4**), that is to say, EWSAT1, LINC00271, and AC105285.1 with the negative association and the other lncRNAs with a positive one. Gene sets associated with proliferation (E2F targets, Myc targets V1 and V2) were overall suppressed in all lncRNAs. Oxidative phosphorylation appeared suppressed in the lncRNAs positively associated with immune gene sets (MAP3K4-AS1, AL049838.1, DNM3OS, MEG3, LINC02544, LINC01711, LINC01638, and AP001189.1).

**Figure 6:**
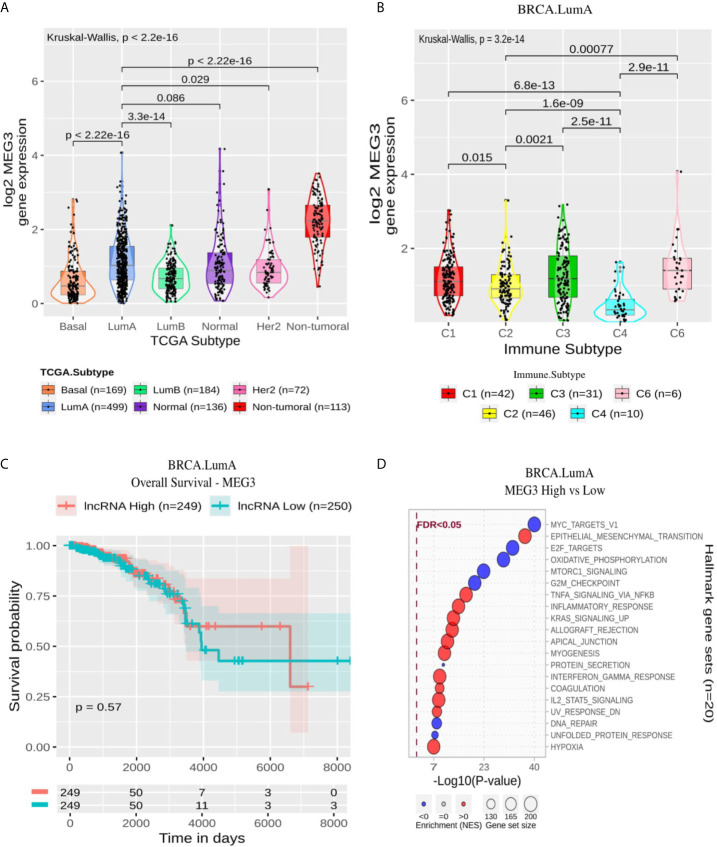
MEG3 panel representation in LumA subtype. **(A)** Box-plots representing MEG3 expression among all breast cancer molecular subtypes. The Kruskal-Wallis test (p-value represented in the panel left top position) was used to differentiate expression between groups, followed by the Wilcoxon test (p-values represented emphasizing comparisons between subtypes). **(B)** Box-plot representing MEG3 in C1,C2, C3, C4, and C6 immune subtypes in LumA patients. **(C)** MEG3 overall survival curves. To define the two groups, MEG3’s median expression classified the patients into “high” and “low” groups. Logrank p-value represented as 0.57. **(D)** Enrichment analysis using Hallmarks gene sets. Red circles refers to activation while blue circles to inactivation. In X axis, a p-value scale is represented. The circle size varies according to the number of genes in the identified gene set.

MEG3 presented a lower expression on BRCA compared with non-tumoral samples and a significant difference when comparing its expression in LumA with other BRCA molecular subtypes except Normal (**Figure 6A**). Within LumA samples, the immune subtypes comparisons resulted in a p-value of 3.2 × 10^−14^ in the Kruskal-Wallis test with a higher expression pattern in C3 and C6. At the same time, C4 stands out with a significantly lower pattern compared with all other immune subtypes (**Figure 6B**). In general, the GSEA analysis resulted in modules associated with immune response enriched with MEG3 overexpression and modules related to proliferation enriched with MEG3 suppression (**Figure 6D**). As expected by the Cox analysis, the Kaplan-Meier did not significantly impact MEG3 expression on OS or PFI (**Figure 6C** and **Supplementary Figure 4C**).

#### Most Specific lncRNAs in LumB Are Associated With Immune Pathways. EBLN3P Is Associated With a Good Prognosis in OS and PFI

LumB specific lncRNAs demonstrated a higher variation in the enriched modules (**Supplementary Table 8** and **Supplementary Figure 3D**). AC009119.1, LINC02620, AL445490.1, and AC104984.4 high expressions were positively related with immune gene sets, while with exception of LINC02620, no clear relation was observed for the proliferation ones. These four lncRNAs were also positively correlated with Leukocyte Fraction (**Supplementary Table 4**). AC092718.3, which did not present a significant correlation with Leukocyte Fraction, was also not related to the enrichment of immune gene sets; otherwise, its enrichment revealed proliferation modules upregulated. USP27X-AS1, AC096733.2, FAM160A1-DT, AL035661.1 and LINC00957 were associated with suppressing gene sets like Allograft Rejection, Interferon Gamma Response and Inflammatory Response; they also related negatively with Leukocyte Fraction (**Supplementary Table 4**).

**Figure 7:**
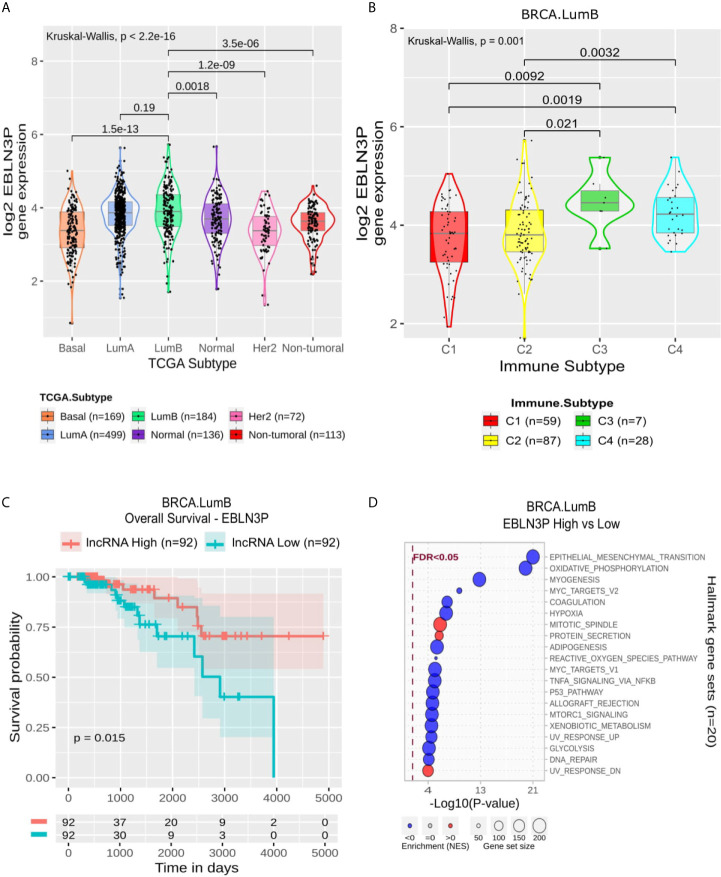
EBLN3P panel representation in LumB subtype. **(A)** Box-plots representing EBLN3P expression among all breast cancer molecular subtypes. The Kruskal-Wallis test (p-value represented in the panel left top position) was used to differentiate expression between groups, followed by the Wilcoxon test (p-values represented emphasizing comparisons between subtypes). **(B)** Box-plot representing EBLN3P in C1,C2, C3, and C4 immune subtypes in LumB patients. **(C)** EBLN3P overall survival curves. To define the two groups, EBLN3P’****s median expression classified the patients into “high” and “low” groups. Logrank p-value represented as 0.015. **(D)** Enrichment analysis using Hallmarks gene sets. Red circles refers to activation while blue circles to inactivation. In X axis, a p-value scale is represented. The circle size varies according to the number of genes in the identified gene set.

EBLN3P is also related negatively to Leukocyte Fraction and with some immune modules. Still, its enrichment revealed a mixed pattern of modules suppressed like Epithelial-Mesenchymal Transition, Oxidative Phosphorylation, Myogenes and Myc Targets (**Figure 7D**). In LumB, its expression was higher in C3 and C4 than C1 and C2 (**Figure 7B**). In comparison, it presented a significantly higher expression in LumB samples than in the other BRCA molecular subtypes except for LumA (**Figure 7A**). In LumB samples, the higher expression of EBLN3P was related to a better outcome in OS but not with PFI in Kaplan-Meier analysis (**Figure 7C** and **Supplementary Figure 4D**).

#### Her2-Specific lncRNAs Are Associated With Immune Activation and XXYLT1-AS2 Associated With Higher PFI

All 10 Her2 specific lncRNAs were related with immune activation as can be seen in their enrichment pattern of Allograft Rejection, Interferon Gamma and Alpha Response, Inflammatory Response, IL6 JAK STAT3 signaling and Complement (**Supplementary Table 9** and **Supplementary Figure 3E**) and in the strong positive correlation with Leukocyte Fraction (ρ range from 0.49 to 0.82 in Spearman’s Correlation, p-adjusted from 1.5 × 10^−5^ to <2.2 × 10^−16^). LINC02384 and LINC02073 are also related to the suppression of proliferation pathways (E2F Targets, G2M checkpoint and MYC Targets).

**Figure 8:**
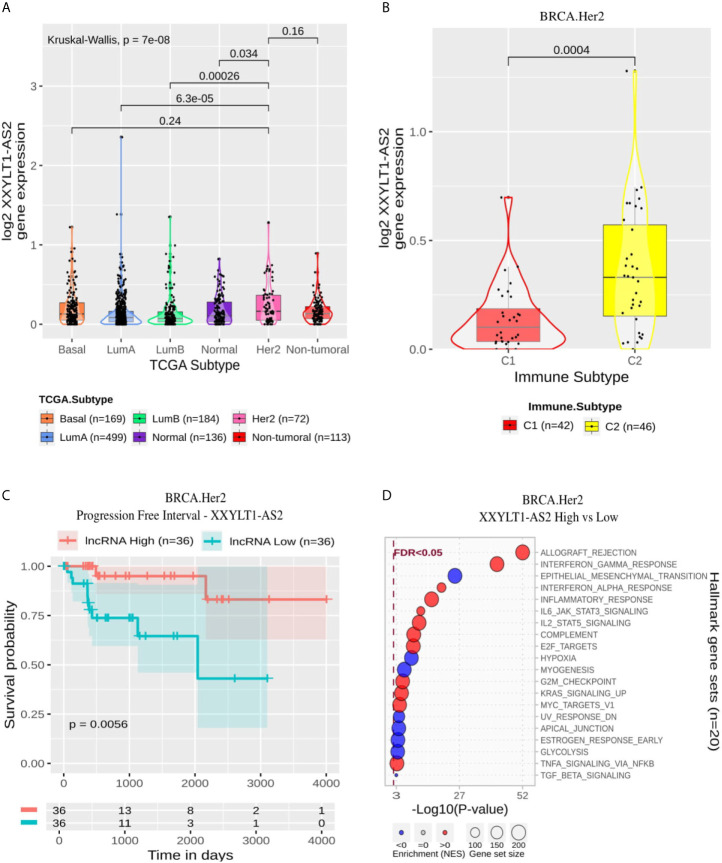
XXYLT1-AS2 panel representation in Her2 subtype. **(A)** Box-plots representing XXYLT1-AS2 expression among all breast cancer molecular subtypes. The Kruskal-Wallis test (p-value represented in the panel left top position) was used to differentiate expression between groups, followed by the Wilcoxon test (p-values represented emphasizing comparisons between subtypes). **(B)** Box-plot representing XXYLT1-AS2 in C1, C2, C3, and C4 immune subtypes in Her2 patients. **(C)** XXYLT1-AS2 progression free interval curves. To define the two groups, XXYLT1-AS2’****s median expression classified the patients into “high” and “low” groups. Logrank p-value represented as 0.0056. **(D)** Enrichment analysis using Hallmarks gene sets. Red circles refer to activation while blue circles to inactivation. In X axis, a p-value scale is represented. The circle size varies according to the number of genes in the identified gene set.

XXYLT1-AS2 showed a significantly higher expression in Her2 than in LumA, LumB and Normal (**Figure 8A**). Within Her2 samples, it was significantly more expressed in the C2 immune subtype (**Figure 8B**) and associated with better prognosis in PFI (**Figure 8C**) but not with OS (**Supplementary Figure 4E**). Its enrichment revealed high positive correlation with Allograft Rejection, Interferon Gamma Response, Inflammatory Response and IL6 and IL2 signaling and negative relation mainly with EMT, Hypoxia and Myogenesis (**Figure 8D**).

## Discussion

Using breast cancer immunogenomics data already published (8), we propose here five distinct immune-related lncRNAs signature according to BRCA molecular subtypes using a two steps SNR selection. Each molecular subtype presented a specific immune-related lncRNAs signature and in GSEA, in general, these lncRNAs functions varied between proliferation and immune activation or suppression, which demonstrates that our selection methodology was able to filter lncRNAs related to the immune response. The survival impact of the selected lncRNAs diverged across the molecular subtypes, in agreement with the fact that the immune activation also differs in terms of prognosis importance between the molecular subtypes (24). For instance, the tumor-infiltrating lymphocytes (TIL) was not related to prognosis in Estrogen Positive BRCA tumors, which may explain the lack of immune-related lncRNAs associated with OS or PFI in LumA/B (24). Nevertheless, most lncRNAs selected for Her2 and Normal were associated with PFI and OS, respectively, reinforcing BRCA molecular subtypes’ well-known heterogeneity. In the article used as a reference (8), the authors elaborated a list of 75 immunomodulatory genes. With this in mind, in **Supplementary Figure 1**, we represented the expression variation (as log2 gene expression z-score) among the molecular subtypes, also considering the immune-related subtype. It is possible to observe a distinct expression pattern in all molecular groups, as expected, regarding breast cancer as a heterogeneous disease. This result reflects the high heterogeneity observed in BRCA molecular subtypes, emphasizing the relevance of characterizing them better molecularly, and we included lncRNA analysis in this complexity.

A specific immune-related signature was proposed to LumA subtype using 11 lncRNAs. Among them, three were suggested related to immune response repression (AC105285.1, LINC00271 and EWSAT1), and none of these were previously investigated under BRCA or immune response aspects (**Supplementary Table 7** and **Supplementary Figure 3C**). However, LINC00271 and EWSAT1 had already been associated with other cancer types. LINC00271 low expression was associated with poor prognosis in papillary thyroid cancer (25) and in adrenocortical tumors (26). EWSAT1 was associated with progression in several cancer types, such as ovarian (27), cervical (28) and colorectal (29).

The lncRNA maternally expressed gene 3 (MEG3) is highlighted here in LumA immune response context. MEG3 is up-regulated in C3 and C6 subtypes and is related to neither overall nor progression aspects (**Figure 6**). The immune subtypes C3 and C6 demonstrate high scores of lymphocyte infiltrate, macrophage regulation and TGF-β response (**Figure 3**). Thus, the increased expression of this lncRNA can be related to immune response activation. Indeed, our functional characterization revealed that MEG3 high expression is associated with several immune hallmarks, such as: “TNFA Signaling Via NFKB,” “Inflammatory Response,” and “Interferon Gamma Response” (**Figure 6D**). MEG3’s role in BRCA immune response is to the best of our knowledge the first time cited here. This lncRNA has only been described in endometrial cancer cells down-regulating *PD-L1* (30). MEG3 is found downregulated in several cancer types, such as BRCA, liver, colorectal and cervical cancer and was experimentally evidenced as *TP53*’*s* regulator (31). In BRCA, MEG3’s downregulation is associated with poor overall survival and tumor staging (32).

In LumB subtype, 11 specific lncRNA were selected, and five of them (LINC00957, AL035661.1, FAM160A1-DT, AC096733.2 and EBLN3P) have their expression related to immune response hallmarks repression (**Supplementary Table 8** and **Supplementary Figure 3D**). These lncRNAs were detected up-regulated in immune subtypes with lower lymphocyte fraction, such as C4. Among these lncRNAs only LINC00957, AL035661.1, and EBLN3P had already been studied in cancer context. LINC00957 high expression was associated with bad prognosis in colorectal cancer (33) and osteosarcoma (34). The lncRNA AL035661.1 was found in a lncRNA profile that managed to efficiently predict early recurrence in hepatocellular carcinoma after curative resection (35) and was associated with EMT in Kidney renal clear cell carcinoma (36).

The lncRNA endogenous Bornavirus-like nucleoprotein 3 (EBLN3P) was highlighted in the LumB subtype. EBLN3P is still not well characterized in the literature since few published studies have focused on its mechanisms and effects on human diseases (37). Dai and colleagues (38) noted that EBLN3P is expressed in osteosarcoma tissues and cell lines. They pointed out that its overexpression promotes proliferation, migration and invasion. Li et al. (37) have already reported EBLN3P as a novel oncogene for liver cancer for similar aspects (37, 38).

EBLN3P’s expression is higher in C3 (Inflammatory) and C4 (Lymphocyte depleted) immune subtypes in LumB samples (**Figure 7B**). In fact, in LumB samples, these immune subtypes exhibit a low TGF-β response score. The role of TGF-β response is still controversial in cancer, depending on the tumor stage. Indeed, TGF-β promotes EMT (39), thus being related to cell proliferation. According to the immune subtypes described by Thorsson et al. (8), the C3 subtype exhibits a low proliferation rate and C4 a moderate one. Using this data with our analysis, we observed a concordance of the data since EBLN3P negatively correlates with TGF-β (**Supplementary Table 8**). In this way, while this lncRNA is highly expressed in these subtypes, TGF-β has a lower expression. The lncRNA EBLN3P’s expression was associated with better survival and disease progression outcomes (**Figure 7C**). Regarding its enrichment analysis in LumB samples, the most significant result was achieved considering “Epithelial Mesenchymal Transition” gene sets. In this scenario, EBLN3P’s low expression is related to EMT activation.

Interestingly, most LumA and LumB lncRNAs were related positively or negatively to the Interferon Gamma Response module. Recently, a study demonstrated that the activation of the interferon signaling pathway in ER+ BRCA relates to resistance to CDK4/6 inhibitors and immune checkpoint activation (40). Understanding the role of lncRNAs in this context may be the subject of future studies.

A total of 10 Basal lncRNAs were selected after SNR analysis. According to functional annotation analysis of these lncRNAs. (**Supplementary Table 5** and **Supplementary Figure 3A**), only the lncRNA APCDD1L-DT’s high expression was related to immune response inhibition. This lncRNA was only evaluated in a competing endogenous RNA network in lung cancer (41), so its immune response regulation role needs to be better understood. The remaining nine lnRNAs were associated with immune response activation (**Supplementary Figure 3A**), and only MIR3142HG was previously studied in cancer context. MIR3142HG polymorphisms were associated with glioma susceptibility and/or prognosis (42). An interesting fact is that MIR3142HG was evidenced as a regulator of IL-1β induced inflammatory response in lung fibroblasts (43). In our analysis, inflammatory response appears to be induced by MIR3142HG (**Supplementary Figure 3A**).

In the Basal, we highlight the lncRNA LINC01871. According to immune subtypes classification, LINC01871 is up-regulated in C2 samples, which is called IFN-γ**** dominant, and its high expression is associated with a better prognosis (**Figure 4**). Our enrichment analysis (**Figure 4D**) showed a strong relation to immune processes. Considering only Basal samples, LINC01871 was associated with “Allograft rejection,” “Interferon Gamma,” “Interferon Alpha,” and “Inflammatory,” for example. In these processes, high expression LINC01871 was associated with the activation of these immune responses. We observe a strong relationship with the leucocyte fraction, which might be related to this observed immune response activation. These results emphasize the relevance of LINC01871 in immune response activation in breast tumor samples, especially in Basal samples, which might be related to better prognosis response.

This lncRNA has already been related to immunity in breast, cervical and gastric cancer. LINC01871 was detected in an immune-related prognosis signature in BRCA and exhibited a strong positive correlation with genes associated with immune response such as *GZMB*, *CTLA4* and *PDCD1*. The authors suggested that this lncRNA may play an important role, mainly related to the above immune processes and immune genes (44). In cervical cancer, LINC01871 was also found in an immune-related prognostic signature being related to immune response and TGF-β signaling pathway (45); additionally, in gastric cancer, LINC01871 expression was positively correlated with CD8+ T cell enrichment levels, cytolytic immune activity, and *CD274* (*PD-L1*) expression levels in TCGA gastric cancer cohort (46).

Taking together our results and background literature is possible to correlate LINC01871 with a cytotoxic immune response. Our enrichment analysis showed an association with Interferon alpha and gamma, which are strongly related to cytotoxic response, going in line with what was observed in other studies (44, 46). In BRCA, CD8+ T cells are reported as prognostic significance in estrogen receptor (ER)-negative BRCA, but not in ER-positive cases, being associated with better clinical outcomes survival and response to treatment (47). Also, CD8+ responses have significantly elevated expression of multiple immune checkpoint molecules, such as programmed cell death 1 (PD-1), programmed death-ligand 1 (PD-L1) and 2 (PD-L2) and cytotoxic T-lymphocyte-associated protein 4 (CTLA4) (48). In this way, a stronger infiltration of CD8+ T cells can predict patient response to standard of care chemotherapy and immune checkpoint blockade therapy, such as anti-PD-1 (49).

According to **Figure 3**, it is possible to observe a distinct pattern of expression of LINC01871, both considering immune subtype and Interferon Gamma response. Recently, Interferon Gamma low expression level was associated with worse prognosis in Basal patients (50). In our analysis, LINC01871 high expression was associated with better prognosis and Interferon Gamma response activation (**Figure 4D**), reinforcing the fact that LINC01871 can be used as a good prognosis marker for Basal patients. This can also be evaluated focusing on therapy response, mainly on immunotherapy. Recently, a combination of an immunotherapy drug with chemotherapy was approved for metastatic triple‐negative BRCA (51); however, due to this group’s high heterogeneity, only a fraction of patients respond well to this treatment. Yang and colleagues (52) defend that an immunity score may be used together with PD-L1 expression to a better design for trials testing immune-checkpoint inhibitors. In this context, lncRNAs like LINC01871 may be used to enhance this selection criterion. However, to be fully applicable as a biomarker, the molecular aspects by which LINC01871 is involved in the immune system activation process need to be better understood.

In the Her2 subtype, ten lncRNAs were evidenced using our selection methodology. All of these lncRNAs were related to immune response activation, as represented in **Supplementary Table 9** and **Supplementary Figure 3E**. Among the ten lncRNAs, four of them had already been related to cancer. LINC02446 is associated with prognosis (53) and EMT activation (54) in bladder cancer. Similarly, USP30-AS1 is associated with prognosis in bladder cancer (54). This lncRNA was also associated with autophagy in ovarian cancer (55), and was related to immune response in cervical cancer (56) and glioblastoma (57). AL365361.1 was associated with good prognosis and immune response in head and neck squamous cell carcinoma (58) and with early recurrence in hepatocellular carcinoma (35). The lncRNA LINC01857 was related to progression in gastric cancer (59), glioma (60), BRCA (61) and B-cell Lymphoma (62). Thus, this lncRNA can be suggested to act as an oncogene. We highlight the lncRNA XXYLT1-AS2, which was associated with progression-free survival in Cox proportional hazard ratio analysis (HR = 0.0011; p-adjusted = 4 × 10^−2^). XXYLT1-AS2 is up-regulated in C2 subtype (**Figure 8B**), and its high expression is related to better progression-free interval (p-logrank = 5.6 × 10^−3^). According to our functional annotation analysis, XXYLT1-AS2 is associated with immune response activation and EMT repression (**Figure 8D**). This result converges with what we observed concerning the progression-free interval since EMT is one of the main pathways activated during the disease progression process. XXYLT1-AS2 is, to the best of our knowledge, the first time described associated with cancer. This lncRNA was only evaluated in Human umbilical vein endothelial cells (HUVECs), and its up-regulation was related to the inhibition of these cell’s proliferation and migration (63).

Finally, for the Normal subtype, a signature composed of 11 lncRNAs was predicted. Most of them are associated with immune response activation and overall survival, and we discuss here LINC02613. This lncRNA is up-regulated in the C3 subtype (**Figure 5B**) and is significantly related to the patient’s overall survival. LINC02613’s high expression is associated with a better prognosis (p-logrank = 7.5 × 10^−3^). The enrichment analysis evidenced that LINC02613 is related to immune response activation and mainly involves cell cycle repression (**Figure 5D**). Indeed, the C3 subtype was described as one with the lowest proliferation rates (8). LINC02613 has not been described in any biological context yet. So we emphasize here the relevance to better characterize this lncRNA.

Considering all the generated and discussed data in this work, we highlight the applied methodology’s significance to look for immune-related lncRNAs. The filtering steps followed by functional annotation efficiently got lncRNAs that might be related to immune response. The role of lncRNAs in regulating immune response is increasingly being explored. Our study is limited to *in silico* analysis; however it brings up new lncRNAs candidates as it is a hypothesis generator article.

Another aspect that is important to discuss here is that we found distinct lncRNAs signature for each molecular subtype that may help find important lncRNAs in the immune response process that may be used to guide therapy candidates or as biomarkers; also our results point out that different lncRNAs may be implicated in immune response depending on BRCA molecular subtypes. Our findings are in agreement with what has been discussed about the heterogeneity of BRCA. We identified that different lncRNAs in each molecular subtype are related to the immune system activation. For example, MEG3 and LINC01871 were associated with activation of Interferon Gamma, in LumA and Basal, respectively. This finding highlights the importance of molecularly characterizing each subtype in order to enable increasingly personalized therapeutic approaches.

Nonetheless, the lncRNAs presented here certainly do not cover all important immune-related lncRNAs in BRCA. Our focus was to find lncRNAs that, in some order, could play a significant role in the immune distinction for each molecular subgroup.

## Conclusion

In conclusion, we present a BRCA specific molecular subtype immune-related lncRNAs signature that may guide future studies aiming to look for important biomarkers in BRCA and highlight the relevance of lncRNAs in the immune subtype’s classification.

## Data Availability Statement

All scripts, datasets, software and algorithms used to generate results, figures, and tables for this study are available on the GitHub repository (https://github.com/sysbiolab/Sup_Material_Mathias2021) and **Supplementary Table 10**.

**Table:** 

DATA SETS.				
RESOURCE	SOURCE			IDENTIFIER
**Normalizad transcriptome data for TCGA BRCA cohort**	XenaBrowser			https://xenabrowser.net/datapages/
**Leukocyte fraction, immune characteristics, and classification for TCGA samples**	Thorsson et al., 2018 (8)			https://www.cell.com/cms/10.1016/j.immuni.2018.03.023/attachment/1b63d4bc-af31-4a23-99bb-7ca23c7b4e0a/mmc2
**List of immune modulators (used for** **Supplementary Figure 1** **)**	Thorsson et al., 2018 (8)			https://www.cell.com/cms/10.1016/j.immuni.2018. 03.023/attachment/8d3ffc74-4db4-4531-a4ad-389dfc8bb7ec/mmc7.xlsx
**Hallmark gene sets**	GSEA-MSigDb v7.2, Liberzon et al. (2015) (22, 23)			https://data.broadinstitute.org/gsea-msigdb/msigdb/release/7.2/msigdb_v7.2.xml
**SOFTWARE AND ALGORITHMS**				
**RESOURCE**		**SOURCE**	**IDENTIFIER**	
**biomaRt v 2.46.3**		Durinck et al., 2005 (16)	https://bioconductor.org/packages/release/bioc/html/biomaRt.html	
**Bioconductor**		n/a	http://www.bioconductor.org/	
**ComplexHeatmap v2.6.2**		Gu et al., 2016 (17)	http://www.bioconductor.org/packages/devel/bioc/html/ComplexHeatmap.html	
**fgsea v1.16.0**		Korotkevich et al., 2019 (21)	https://bioconductor.org/packages/release/bioc/html/fgsea.html	
**survival v3.2-10**		Therneau, 2020 (20)	https://cran.r-project.org/web/packages/survival/index.html	
**survminer v0.4.8**			http://www.sthda.com/english/rpkgs/survminer/	

## Author Contributions

CM, JM, and BA conceived the presented idea. CM, JM, and BA developed the theory and performed the computations. DG, MC, and JO verified the analytical methods. DG, MC, and JO supervised the findings of this work. All authors contributed to the article and approved the submitted version.

## Funding

This work was supported by the Public Research Agencies CAPES (001-Coordenação de Aperfeiçoamento de Pessoal de Nível Superior) and CNPq (Conselho Nacional de Desenvolvimento Científico e Tecnológico) processo 153771/2018-6. JM received scholarship from the BIG DATA innovation program from the Associação Hospitalar de Proteção à Infância Raul Carneiro-AHPIRAC (2020).

## Conflict of Interest

The authors declare that the research was conducted in the absence of any commercial or financial relationships that could be construed as a potential conflict of interest.

## Supplementary Material

Supplementary Figure 1 Heatmap of Immune modulators gene set (n=74) proposed by the reference article Thorsson et al. (8) in breast cancer molecular subtypes. For color gradient, maximum and minimum column-wise z-scores were set to +2 and −2 respectively. Each column represents a sample and were semi-supervised clustered within the molecular subtypes.

Supplementary Figure 2 Venn diagram representing specific and shared immune related lncRNAs in breast cancer (BRCA) molecular subtypes after filtering for 0.90 quantile in signal to noise ratio (SNR) for the BRCA molecular subtypes. 593 lncRNAs were filtered by this criterion for each molecular subtype.

Supplementary Figure 3 Enrichment map for immune-related lncRNA signature for each molecular subtype: **(A)** Basal, **(B)** LumA, **(C)** LumB, **(D)** Normal, and **(E)** Her2. The lncRNAs are organized in the X axis and Hallmarks gene sets in the Y axis. Red circles refer to positive enrichment scores while blue circles to negative. The circle size varies according to -log10 (adjusted p-value), bigger circles mean greater significance.

Supplementary Figure 4 Kaplan-Meier analysis for overall survival for LINC01871 in Basal **(A)** and XXYLT1-AS2 in Her2 **(E)** and for Progression Free Interval for MEG3 in LumA **(B)**, EBLN3P in LumB **(C)** and LINC02613 in Normal **(D)**. The groups of High and Low expressions are based on the median value of its respective lncRNA expression. Confidence interval and logrank p-value are shown and values below 0.05 are considered significant.

Supplementary Table 1 Breast cancer cohort stratification into the immune subtypes.

Supplementary Table 2 Ensembl and HGNC Symbol from lncRNAs used in this study.

Supplementary Table 3 First and second SNR calculated for lncRNAs for each molecular subtype.

Supplementary Table 4 Cox Proportional Hazard models for the specific immune-related lncRNAs and Leukocyte correlation.

Supplementary Tables 5–9 GSEA Hallmarks analysis for the specific immune-related lncRNAs organized by the molecular subtype.

Supplementary Table 10 Data sets, software and algorithms used in this study.
