# Supplementary figures and images for: Unraveling Immune-Related lncRNAs in Breast Cancer Molecular Subtypes
Source: Front Oncol. 2021 May 31;11:692170. doi: 10.3389/fonc.2021.692170 (PMC8202402; doi:10.3389/fonc.2021.692170)

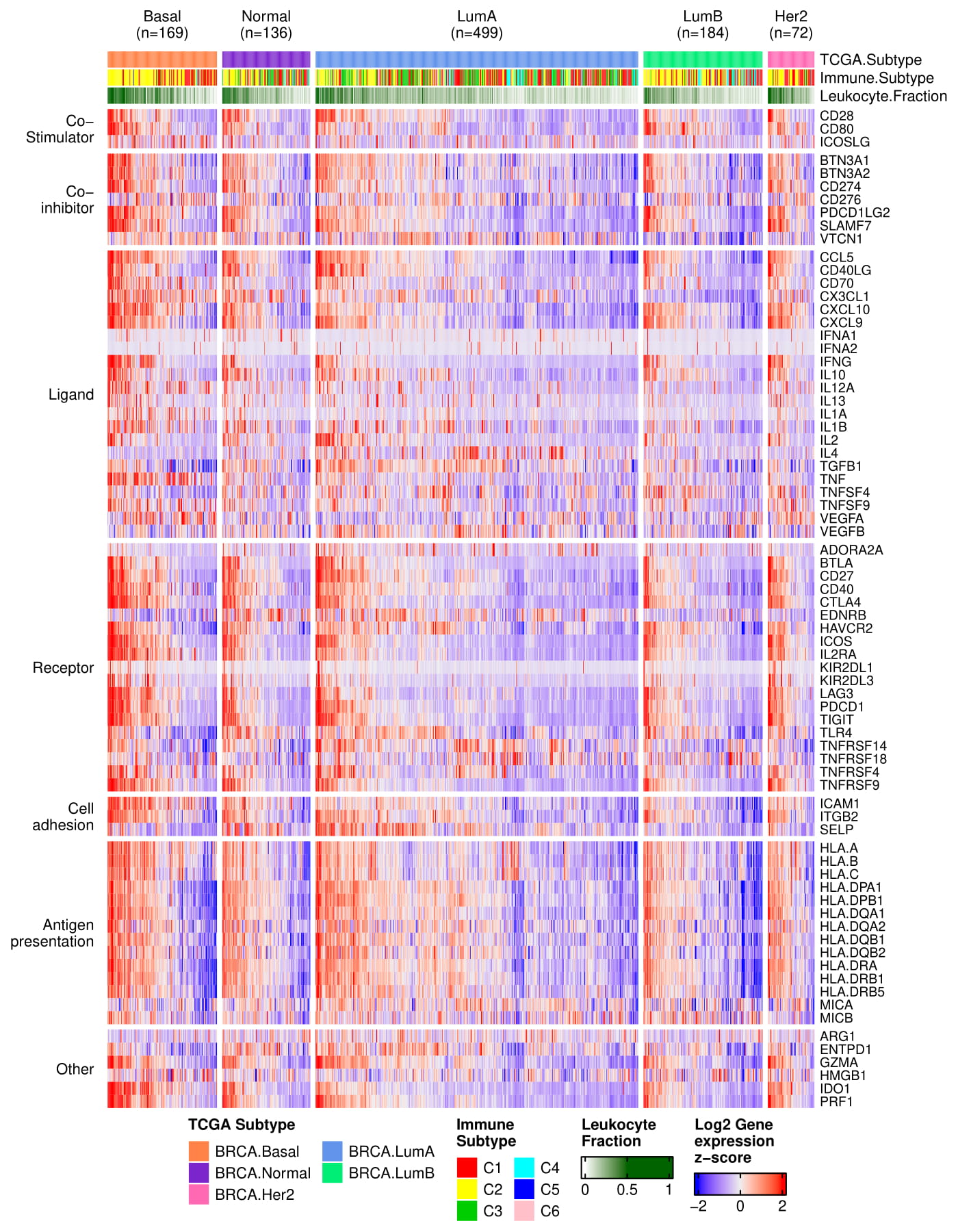

Supplement: Supplementary Figure 1 — Heatmap of Immune modulators gene set (n=74) proposed by the reference article Thorsson et al. (8) in breast cancer molecular subtypes. For color gradient, maximum and minimum column-wise z-scores were set to +2 and −2 respectively. Each column represents a sample and were semi-supervised clustered within the molecular subtypes. [file Image_1.jpeg]

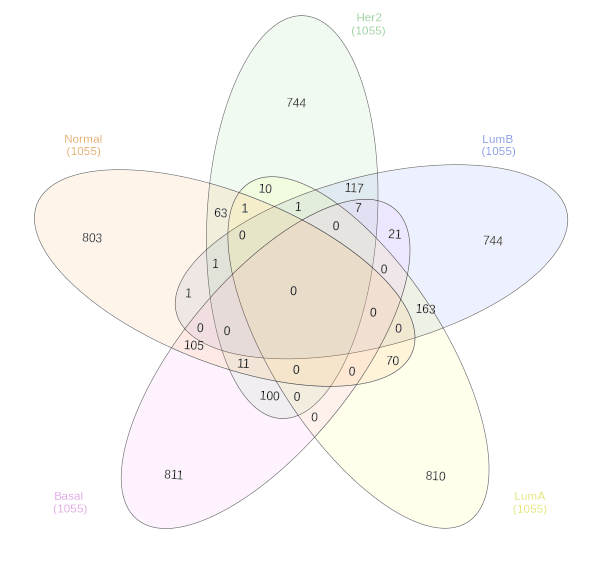

Supplement: Supplementary Figure 2 — Venn diagram representing specific and shared immune related lncRNAs in breast cancer (BRCA) molecular subtypes after filtering for 0.90 quantile in signal to noise ratio (SNR) for the BRCA molecular subtypes. 593 lncRNAs were filtered by this criterion for each molecular subtype. [file Image_2.jpeg]

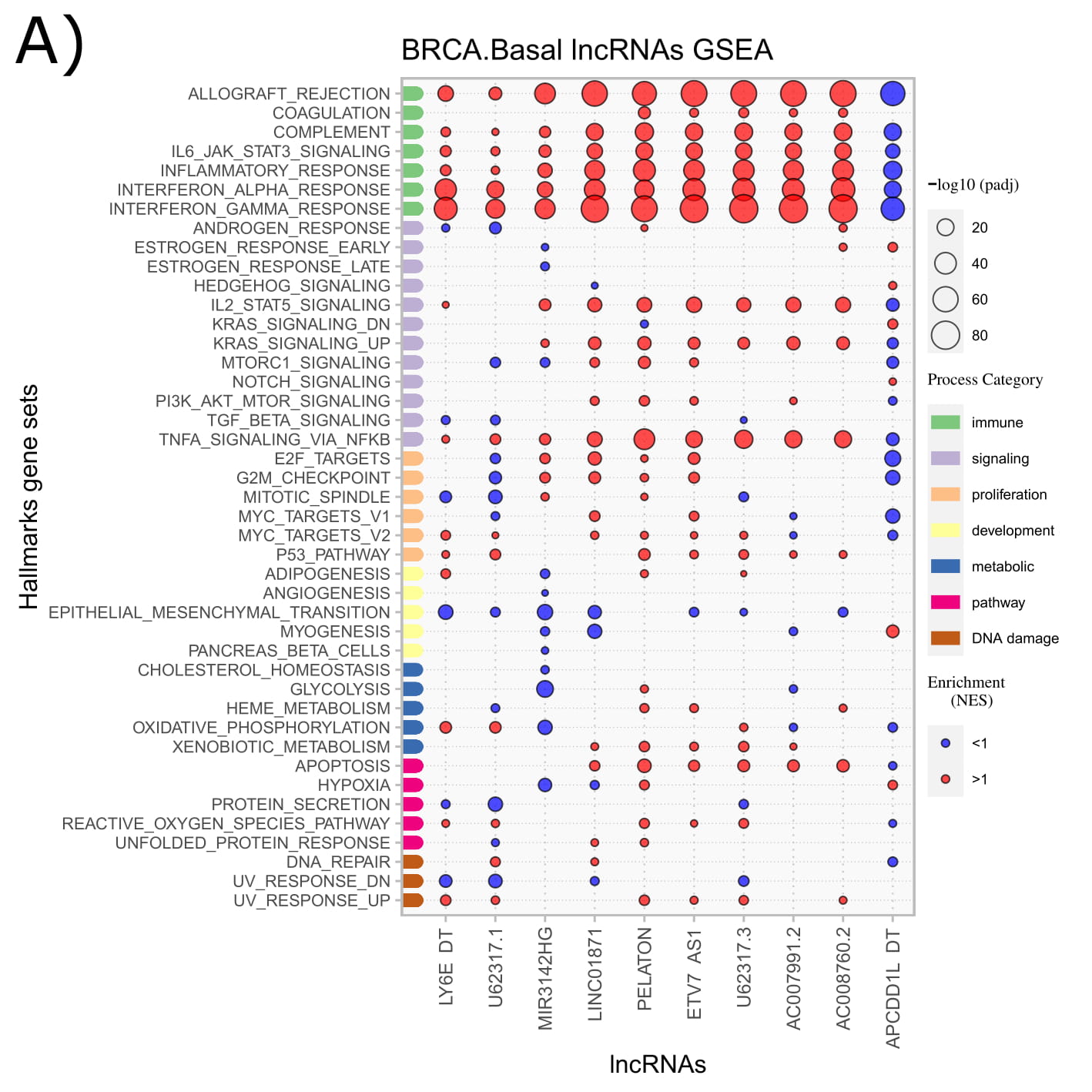

Supplement: Supplementary Figure 3 — Enrichment map for immune-related lncRNA signature for each molecular subtype: (A) Basal, (B) LumA, (C) LumB, (D) Normal, and (E) Her2. The lncRNAs are organized in the X axis and Hallmarks gene sets in the Y axis. Red circles refer to positive enrichment scores while blue circles to negative. The circle size varies according to -log10 (adjusted p-value), bigger circles mean greater significance. [file Image_3.jpeg]

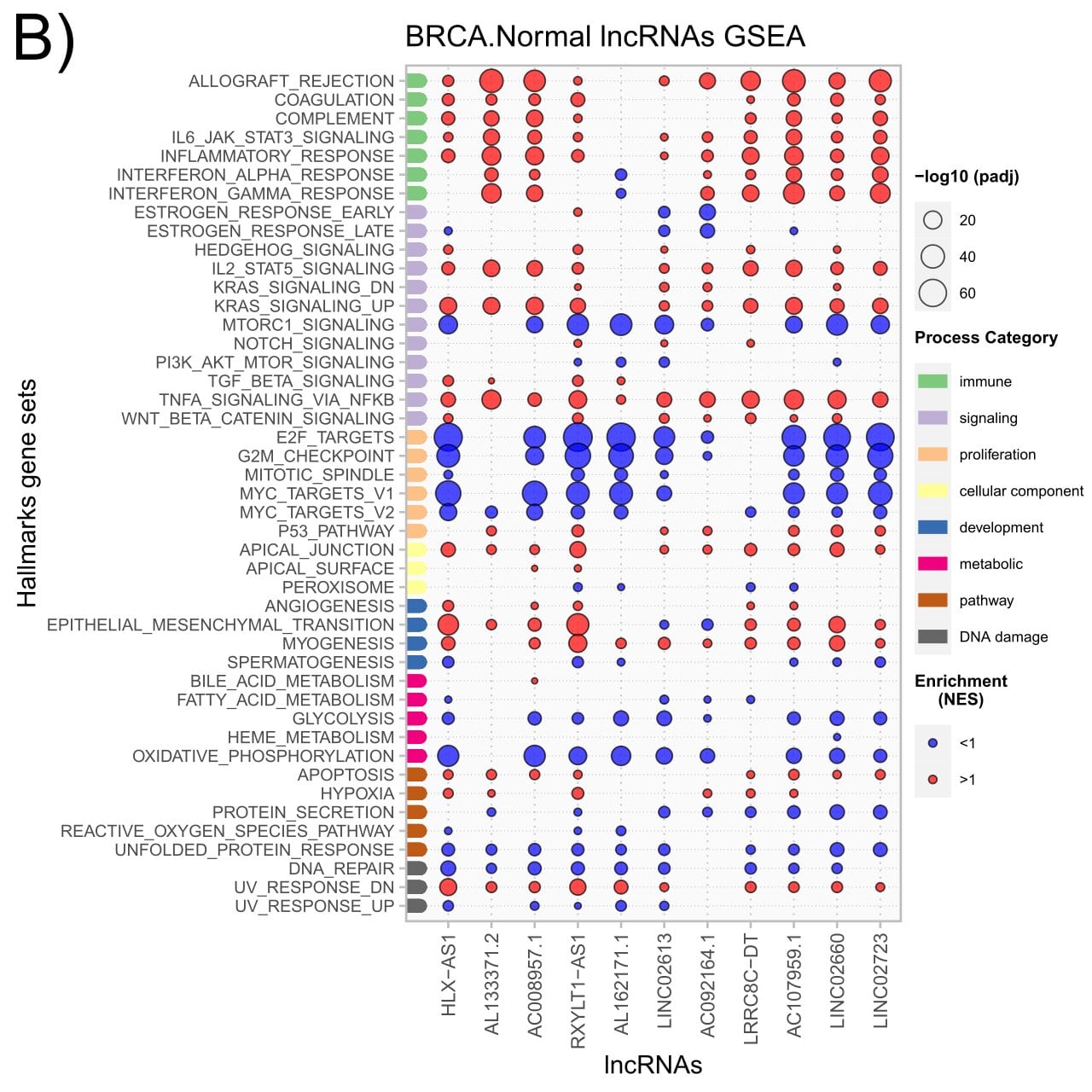

Supplement: Supplementary Figure 4 — Kaplan-Meier analysis for overall survival for LINC01871 in Basal (A) and XXYLT1-AS2 in Her2 (E) and for Progression Free Interval for MEG3 in LumA (B), EBLN3P in LumB (C) and LINC02613 in Normal (D). The groups of High and Low expressions are based on the median value of its respective lncRNA expression. Confidence interval and logrank p-value are shown and values below 0.05 are considered significant. [file Image_4.jpeg]

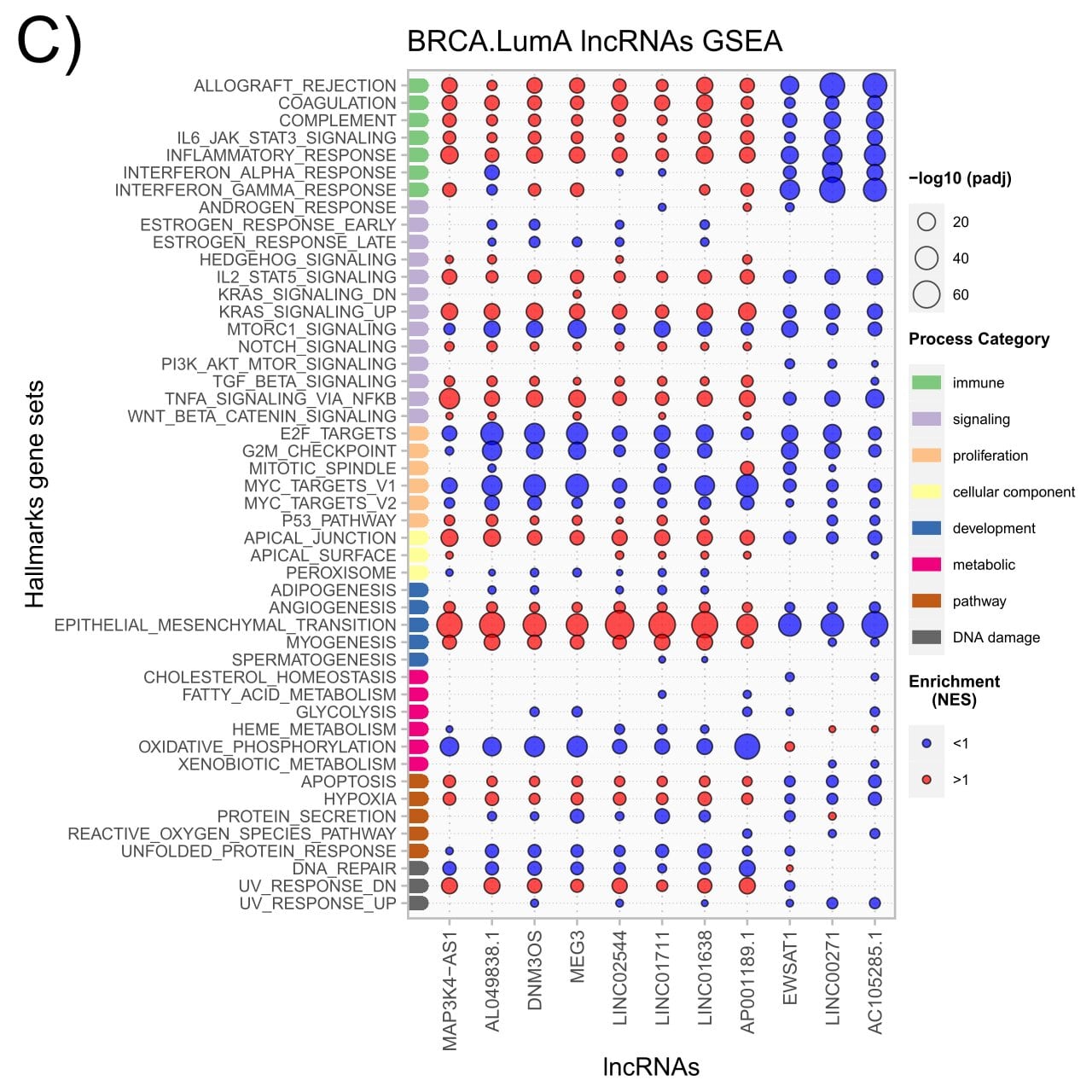

Supplement: Supplementary file 5 [file Image_5.jpeg]

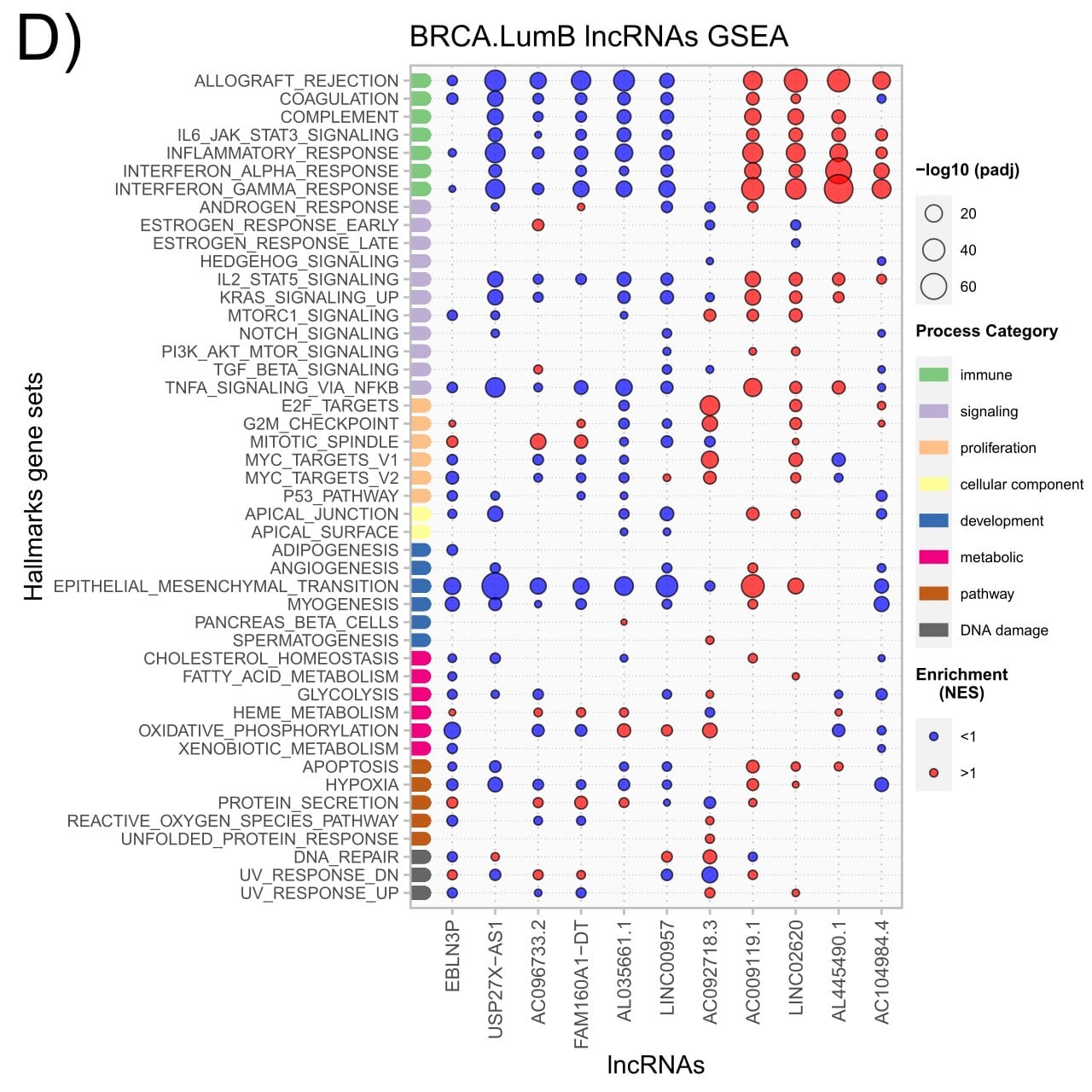

Supplement: Supplementary file 6 [file Image_6.jpeg]

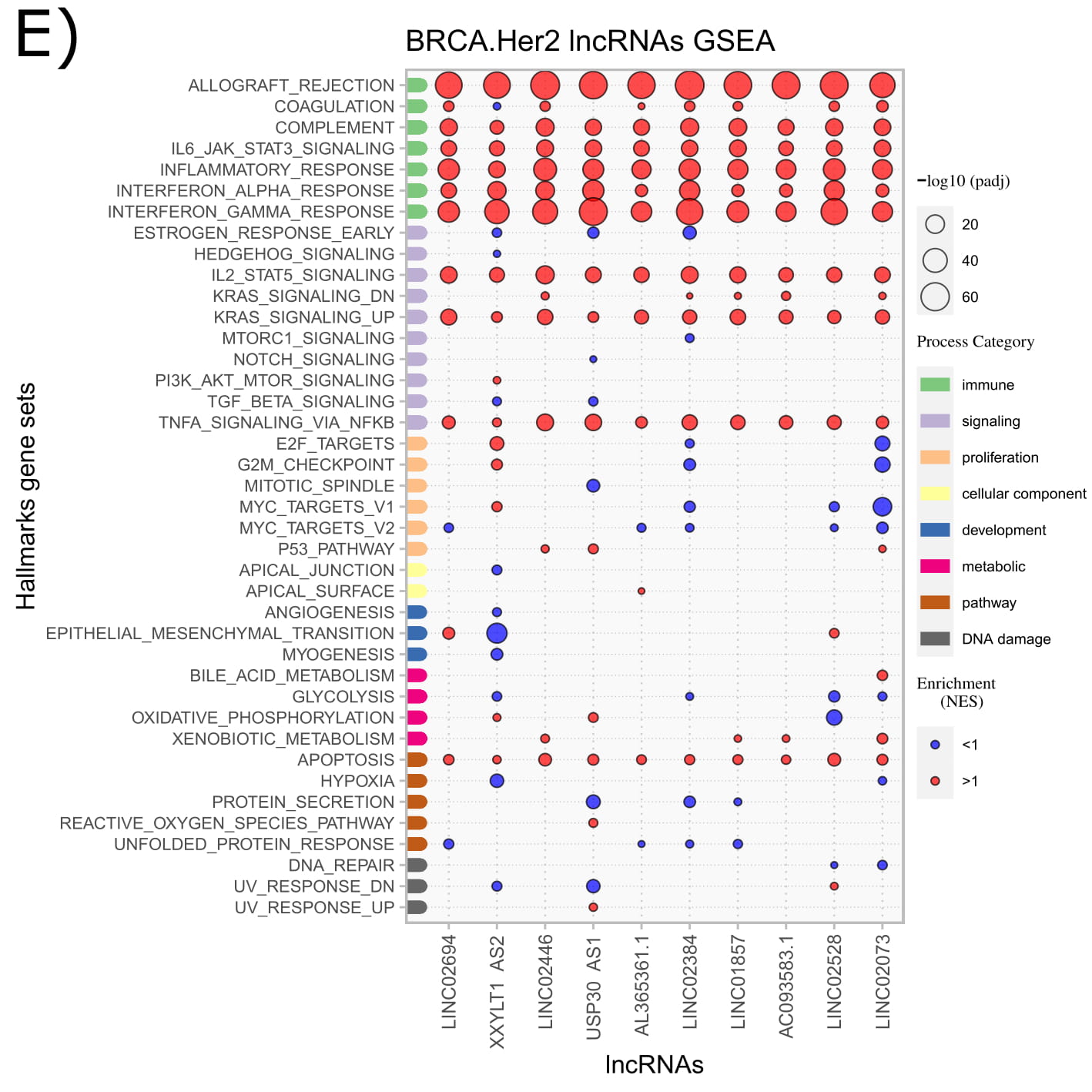

Supplement: Supplementary file 7 [file Image_7.jpeg]

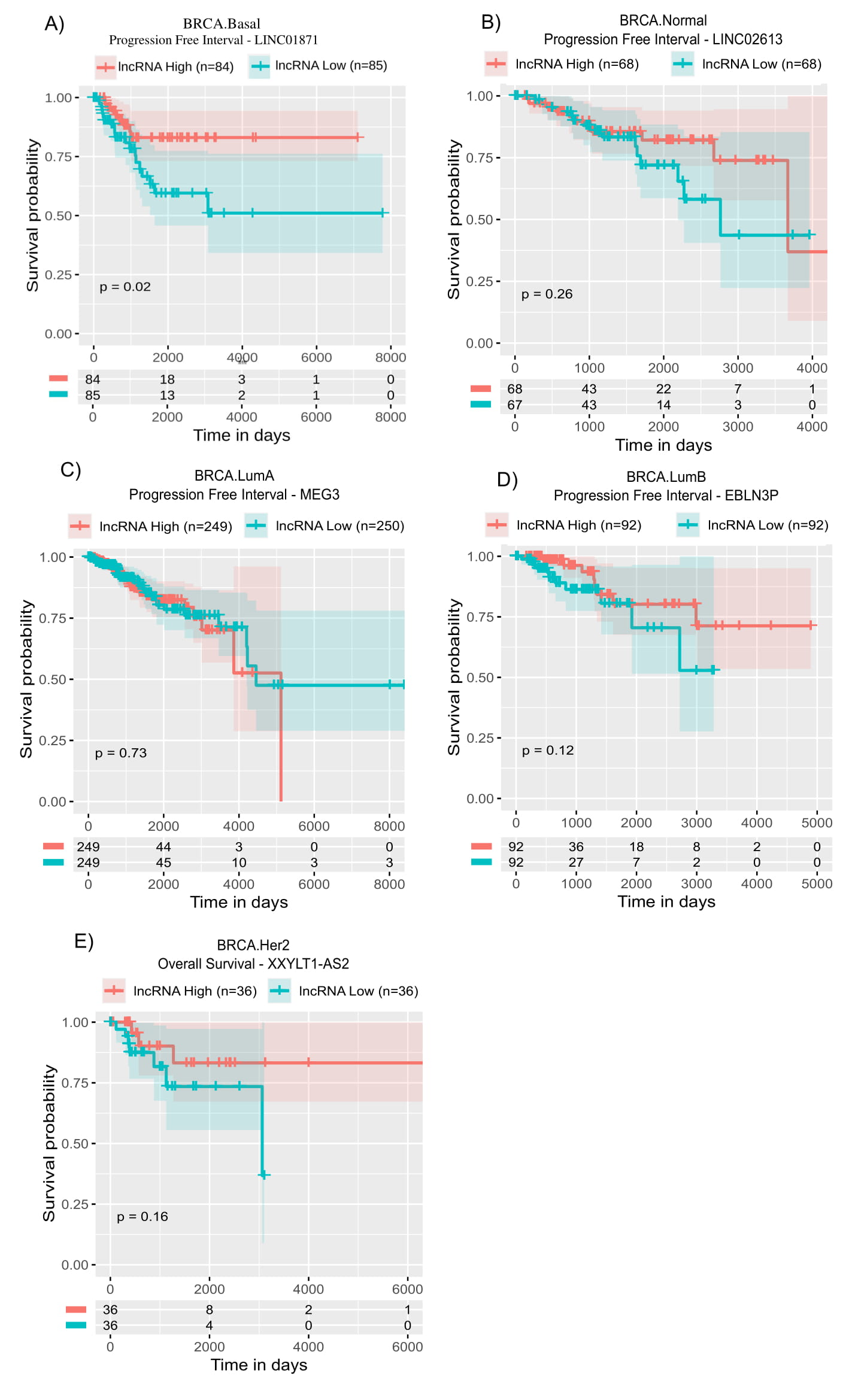

Supplement: Supplementary file 8 [file Image_8.jpeg]
